# Modern Anesthetic Ethers Demonstrate Quantum Interactions with Entangled Photons

**DOI:** 10.1038/s41598-019-47651-1

**Published:** 2019-08-05

**Authors:** Ryan K. Burdick, Juan P. Villabona-Monsalve, George A. Mashour, Theodore Goodson

**Affiliations:** 1 Department of Chemistry, University of Michigan, Ann Arbor, MI 48109 USA; 2 Center for Consciousness Science, Department of Anesthesiology, University of Michigan Medical School, Ann Arbor, MI 48109-5048 USA

**Keywords:** Biophysics, Neuroscience, Chemistry, Physics

## Abstract

Despite decades of research, the mechanism of anesthetic-induced unconsciousness remains incompletely understood, with some advocating for a quantum mechanical basis. Despite associations between general anesthesia and changes in physical properties such as electron spin, there has been no empirical demonstration that general anesthetics are capable of functional quantum interactions. In this work, we studied the linear and non-linear optical properties of the halogenated ethers sevoflurane (SEVO) and isoflurane (ISO), using UV-Vis spectroscopy, time dependent-density functional theory (TD-DFT) calculations, classical two-photon spectroscopy, and entangled two-photon spectroscopy. We show that both of these halogenated ethers interact with pairs of 800 nm entangled photons while neither interact with 800 nm classical photons. By contrast, nonhalogenated diethyl ether does not interact with entangled photons. This is the first experimental evidence that halogenated anesthetics can directly undergo quantum interaction mechanisms, offering a new approach to understanding their physicochemical properties.

## Introduction

Inhalational anesthetics were first used more than 170 years ago, when simple aliphatic ethers, such as diethyl ether, were commonly used to induce unconsciousness. Most inhalational anesthetics in clinical use today are halogenated ethers, such as sevoflurane (SEVO) and isoflurane (ISO), which can be considered derivatives of the anesthetic diethyl ether. To date, a description of the mechanism of anesthetic-induced unconsciousness has been approached from the perspective of classical chemistry interactions between anesthetics and neuronal compounds on the macroscopic level^1^. This approach has shown how anesthetics can target particular proteins and protein-based compounds to manipulate the conformation of these compounds found in neurochemical synapses, leading to changes in the chemical potential of the surrounding environment. However, other studies have suggested that anesthetics may undergo a quantum interaction mechanism to induce unconsciousness^2–5^, but—unlike the foundational empirical studies identifying binding between anesthetics and protein targets—it is unclear if general anesthetics can interact directly with quantum systems, including those involving long-distance entanglement. In order for a quantum interaction mechanism to be possible, two initial conditions would have to be met: (1) the anesthetic molecules must have the ability to target particles much smaller than the macroscopic compounds previously studied, as only atomic and subatomic particles will strongly adhere to the unique behaviors of quantum mechanics; and (2) the interactions cannot be explained using classical mechanics. Two previous studies have shown how quantum properties may play a role in anesthetic-induced unconsciousness. Turin *et al*.^6^ reported electron spin changes in fruit flies that occurred after they were given anesthetics, and Li *et al*.^7^ reported that xenon isotopes without nuclear spin were more potent anesthetics than isotopes with nuclear spin. Although both of these studies indirectly suggest that quantum mechanics might relate to the mechanism of general anesthesia, neither study investigated whether anesthetics could directly interact with a quantum system.

Previous physical, pharmacological, and neuroscientific studies have not been able to describe completely the mechanism of anesthetic action for SEVO and ISO. Despite studies of SEVO’s and ISO’s NIR/IR optical properties^8–17^, as well as ground state chemical structures and properties^10,18–29^, very little is known about their linear and nonlinear optical properties in the visible and UV light range and their excited state electronic properties. Although the optical properties of anesthetics have not been studied in an attempt to understand their ability to change neuronal functioning and induce unconsciousness, spectroscopy experiments have been completed on neurons to produce action potentials using only light. Hirase *et al*.^30^ used high intensity pulsed IR light to trigger action potentials in pyramidal neurons. Although this experiment with high energy laser light damaged the cells, Wells *et al*.^31^ performed a similar experiment using IR light with much lower incident laser power and triggered action potentials in neurons *in vivo*, attributed to a photothermal effect^32^, without damaging the cells. The low light intensity required to trigger the action potentials shows the high sensitivity of neurons to light. In addition to these experimental light sources, biophotons, i.e. light emitted by cells during specific metabolic processes^33^, have been identified in neural tissue and hypothesized as auxiliary carriers in neuronal information transfer^34–38^. Thus, the study of photons is justified in the context of neural function and anesthetic-induced disruption of information processing

In this study we investigated the direct interaction between anesthetics and a quantum system using entangled two-photon spectroscopy. This spectroscopy technique utilizes pairs of photons whose quantum states are strongly correlated such that they must be treated as one body (i.e., they are entangled). The interaction mechanisms of a molecule with entangled photons vs classical photons are different, as shown in previous work by Lee *et al*.^39^. Therefore, both interaction processes must be investigated for a complete understanding of the optical properties of the molecule. Using 800 nm photons in a classical two-photon spectroscopy experiment, neither SEVO nor ISO interacted with the classical photons despite using very high light intensity (i.e., large number of photons) in our experiment. However, SEVO and ISO did interact with the 800 nm entangled photons at extremely low light intensity. By contrast, diethyl ether (a nonhalogenated ether used for reference) did not, showing a unique and unexpected sensitivity to a quantum correlated system in association with halogenation.

## Results

### Linear optical properties

**Figure 1:**
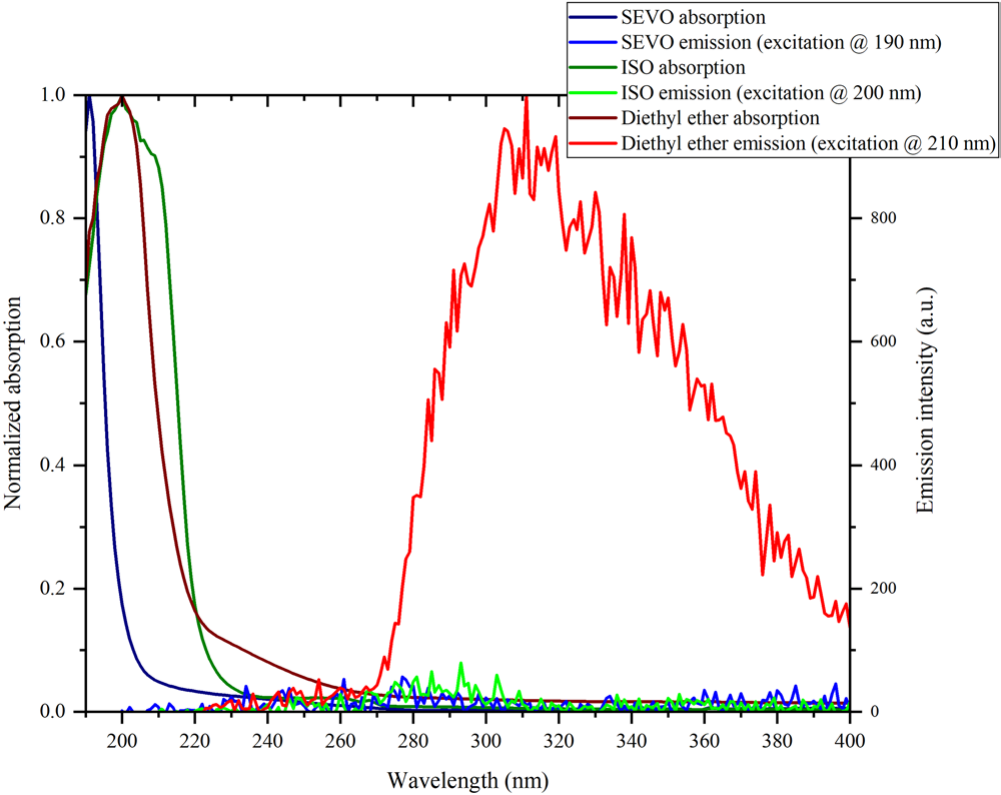
UV absorption and emission spectra of sevoflurane, isoflurane and diethyl ether in liquid phase.

Previous studies of steady-state UV absorption of SEVO and ISO were completed in the gas phase^40,41^. It was stated that SEVO did not have any absorption above 200 nm, and no absorption spectrum was shown^40^. ISO had an UV absorption onset at 215 nm and continued to increase down to 200 nm^40^, with another report showing the UV absorption continuing to increase down to 120 nm^41^. To the best of our knowledge, the UV absorption spectra for SEVO and ISO in Fig. 1 are the first reported absorption spectra for ISO in the liquid phase and for SEVO in any phase. The absorption spectrum for diethyl ether, an aliphatic ether with a similar structure as SEVO and ISO, is shown for comparison. Fig. 1 also shows the emission spectra of SEVO and ISO when excited at their respective *λ*_max_ (while 190 nm may not be the true *λ*_max_ of liquid SEVO, it was the lowest wavelength available from our spectrophotometer and thus treated as *λ*_max_ for emission experiments). The emission spectrum for diethyl ether, excited at 210 nm, is also shown for comparison.

The absorption spectrum for ISO peaks at 200 nm, the same as diethyl ether, while the absorption spectrum of SEVO is blue-shifted and continues to increase down to 190 nm without peaking. No absorption was shown for either compound above 270 nm. Previous studies have shown that fluorinated ethers have an UV absorption that is blue-shifted relative to aliphatic ethers^42,43^, as we see when comparing SEVO to diethyl ether in Fig. 1. However, the fluorinated ether ISO, which also contains a chlorine atom, is not blue-shifted from diethyl ether at all. Although diethyl ether shows broad emission from ~270 nm to beyond 400 nm, peaking around 310 nm (which corresponds well with a previous study that reported an emission peak around 306 nm^44^), neither SEVO nor ISO show any emission above background noise.

### Ground and first excited state electronic properties

Ground and first excited state electronic structure calculations were completed for SEVO and ISO to explain the linear optical properties in Fig. 1. In order to understand the effect that the halogens in SEVO and ISO have on the molecular orbitals and energy levels, calculations were also completed for aliphatic ethers that have the same structure as the halogenated ethers but with all halogens replaced with hydrogens. The aliphatic parent ether of SEVO is methyl isopropyl ether and that of ISO is ethyl methyl ether. The results of the TD-DFT calculations for the ground-state-to-excited-state transitions in all four molecules are summarized in Table 1. Results for diethyl ether are also shown to compare with the experimental linear optical properties obtained in Fig. 1.

**Table 1:** TD-DFT/B3LYP/6–311 + G(2d,p) calculations for SEVO, ISO, their aliphatic parent ethers (methyl isopropyl ether and ethyl methyl ether, respectively), and diethyl ether.

Compound	S_0_ → S_1_ excitation energy (eV/nm)	HOMO-LUMO gap (eV)	S_0_ → S_1_ transition dipole moment (D)	S_0_ → S_1_ oscillator strength	Permanent dipole moment (D)
methyl isopropyl ether	6.307/196.6	7.185	0.6318	0.0096	1.2974
SEVO	8.339/148.7	9.290	0.6905	0.0151	2.2638
ethyl methyl ether	6.368/194.7	7.313	0.9523	0.0219	1.2461
ISO	6.923/179.1	8.324	0.2374	0.0015	1.8675
diethyl ether	6.343/195.5	7.268	0.8621	0.0179	1.1498

**Figure 2:**
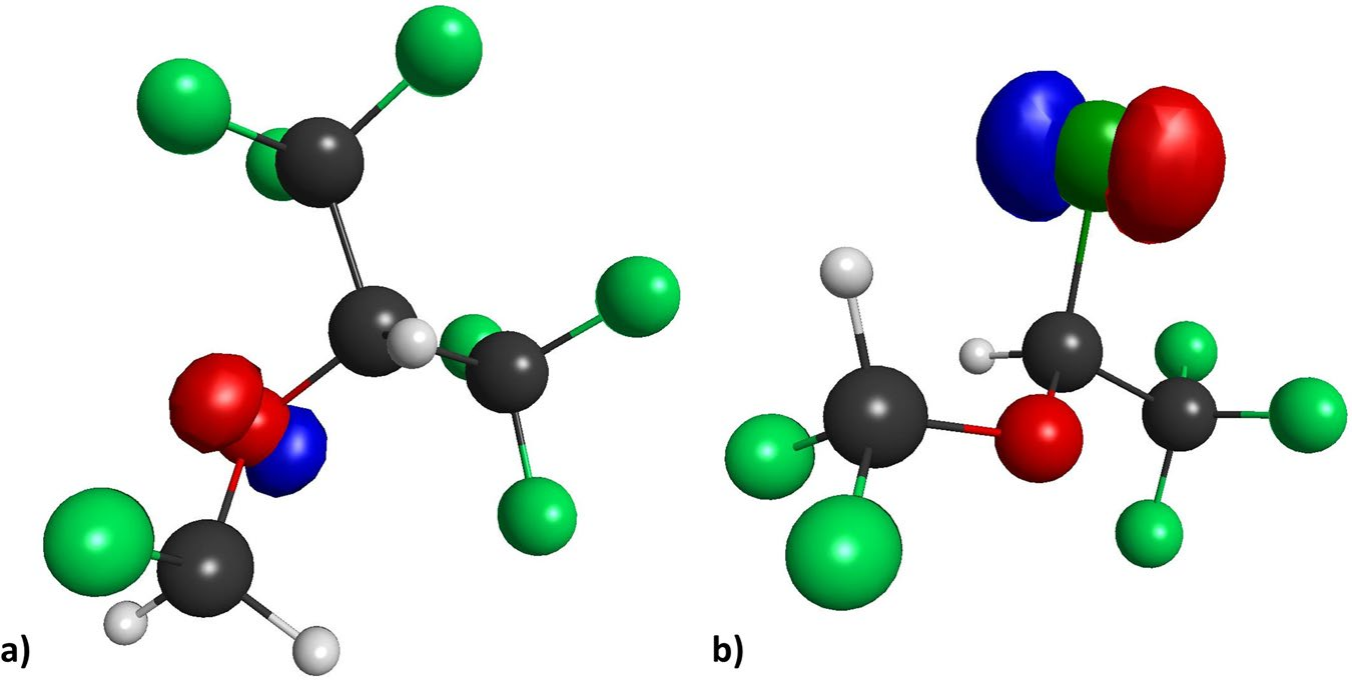
HOMO for (**a**) SEVO, and (**b**) ISO. Isosurface values were chosen such that only the most predominant location of each orbital is shown in order to identify the character of the HOMO.

Similar to the observed blue-shift for SEVO (compared to diethyl ether) in the UV absorption spectra in Fig. 1, the TD-DFT results in Table 1 also show a blue-shift for SEVO in the first excitation energy, corresponding to the ground state (S_0_) to first excited state (S_1_) transition, compared to its aliphatic parent ether, methyl isopropyl ether. A similar blue-shift is observed in ISO compared to its aliphatic parent ether, ethyl methyl ether, though the shift (+0.555 eV) is not as large as compared to SEVO’s blue-shift (+2.032 eV). This correlates well with our UV absorption spectra in Fig. 1, where SEVO has a large blue-shift compared to the aliphatic ether while ISO has little to no blue-shift. This difference between SEVO and ISO is most likely due to the different characteristics of their HOMOs, shown in Fig. 2. The HOMO for SEVO is located predominantly on the oxygen lone pairs, while that of ISO is located predominantly on the chlorine lone pairs.

The excitation wavelength for SEVO in Table 1 (148.7 nm) shows why *λ*_*max*_ is not seen in the UV absorption spectrum in Fig. 1, since the calculated energy to the first excited state in SEVO requires a wavelength beyond the limits of our spectrophotometer. The wavelength of the first excitation energy in Table 1 for diethyl ether (195.5 nm) corresponds very well with the peak in the absorption spectrum for diethyl ether in Fig. 1 (200 nm), thus confirming the accuracy of our electronic structure calculations. The calculated permanent dipole moments are also very close to literature values for diethyl ether^45^, SEVO^18,20,21,27^, and ISO^22^. Additional electronic structure calculation results for molecular orbitals are provided in Supplementary Figs S1–S5.

### Nonlinear optical properties

**Figure 3:**
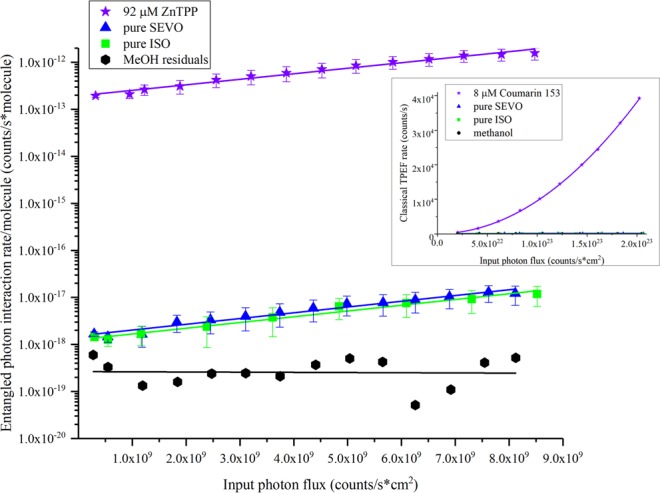
Entangled photon interaction rate per molecule vs input photon rate for sevoflurane and isoflurane, with ZnTPP used as a standard. Classical two-photon results for sevoflurane and Coumarin 153 as a standard are shown in the inset. Error bars were calculated first for the sample and solvent transmission scans separately using the percent standard deviation of 50 single photon count measurements for toluene (a non-interacting solvent) at 4 different incident laser powers. This error was then propagated to calculate the interaction signal error bars.

Since the absorption spectra in Fig. 1 and the theoretical calculations in Table 1 show that the first excited electronic state for both SEVO and ISO is below 200 nm, neither compound was expected to have a two-photon interaction with 800 nm photons through nonlinear optical processes. The classical two-photon interaction properties of pure SEVO and pure ISO were tested by measuring the two-photon excited fluorescence (TPEF) using 800 nm incident light. The results are shown in the inset of Fig. 3, using Coumarin 153 as a standard. The entangled two-photon interaction properties of pure SEVO and pure ISO were studied next, also using 800 nm incident light. The results are shown in Fig. 3, using ZnTPP as a standard. In our entangled two-photon experiments, the transmission of the entangled photons through a blank (solvent) is compared with the transmission through the sample (SEVO and ISO). Any difference between the transmission intensities is due to an interaction between the sample and entangled photons.

**Figure 4:**
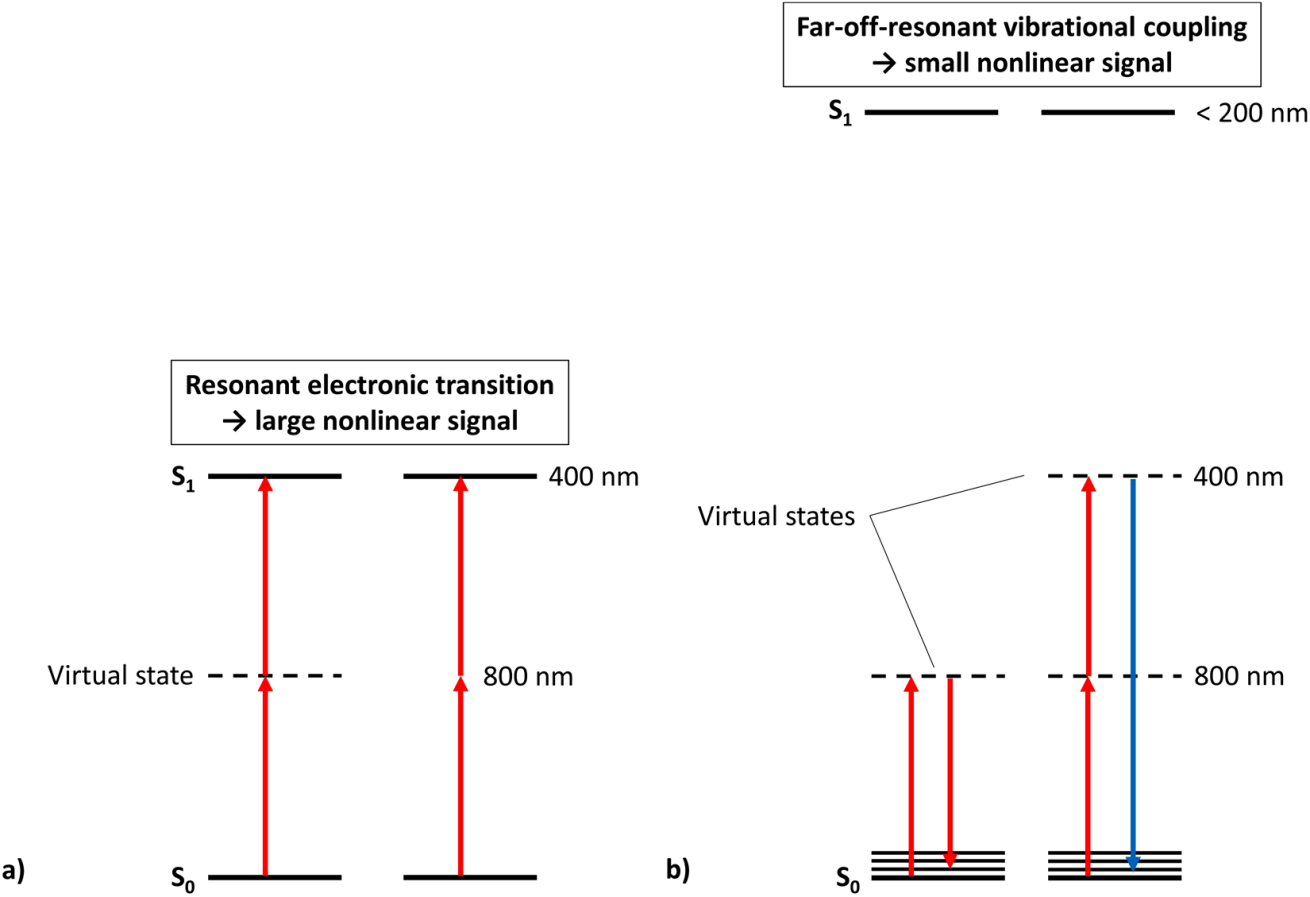
Comparison of interaction mechanisms using entangled photons: (**a**) absorption transitions resonant with excited electronic states, seen in ZnTPP; (**b**) far-off-resonant interactions yielding no electronic excitation, seen in SEVO and ISO.

As expected for the classical two-photon experiment, seen in the inset of Fig. 3, neither SEVO nor ISO interacted with the 800 nm light. Coumarin 153, used as a standard, showed the expected quadratic dependence of TPEF on the input photon flux, validating our experimental method. Surprisingly, the entangled two-photon experiment did show interaction with the 800 nm entangled photons for the SEVO and ISO solutions, seen in Fig. 3. The log scale of the y-axis in Fig. 3 (entangled photon interaction rate per molecule) shows that SEVO and ISO yielded entangled two-photon interaction signals that are 5 and 6 orders of magnitude smaller than that of the standard, ZnTPP, respectively. The difference in signal intensity between the anesthetics and ZnTPP is expected since the molecules undergo different interaction mechanisms, shown in Fig. 4.

For chromophores that can absorb light at 400 nm, like ZnTPP, the two 800 nm photons are resonant with a real, excited electronic state in the molecule, seen in Fig. 4a. When the incoming entangled photons are resonant with a real eigenstate, the molecule can interact with the photons through two pathways (as explained in previous work^46,47^): (1) the virtual state pathway, where the light can create a coupling with an intermediate virtual state, and the molecule will absorb the two photons in a two-step transition through that virtual state; (2) the permanent dipole pathway, where the light can create a coupling between the permanent dipoles of the ground and excited electronic state, and the molecule will absorb the two photons simultaneously. The probability of the virtual state pathway is inversely proportional to the detuning energy of the virtual state, i.e., the difference in energy between the virtual state and the real eigenstates in the molecule. Since this detuning energy is usually quite large (~12,500 cm^−1^), the virtual state pathway has a low probability; but the higher probability of the permanent dipole pathway leads to a large optical signal, as seen in many chromophores studied previously^39,46,48–50^, including ZnTPP.

**Figure 5:**
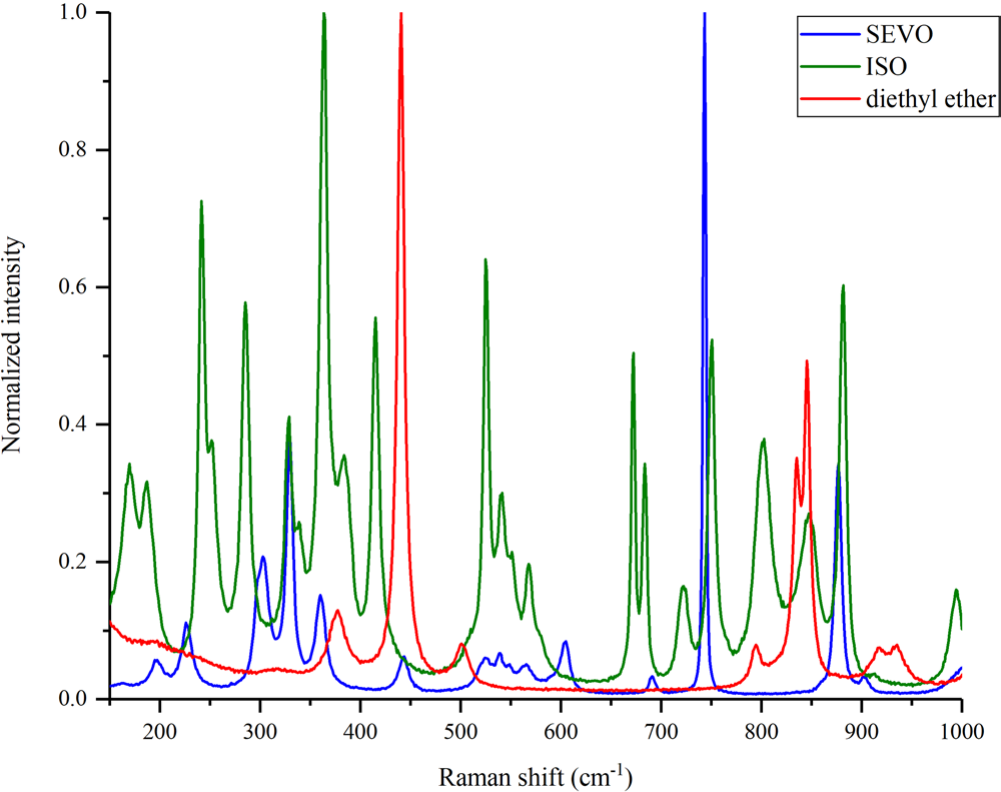
Raman spectra of SEVO, ISO, and diethyl ether with excitation of 785 nm.

For SEVO and ISO, the 800 nm entangled photons are far-off-resonance with the first excited electronic state, shown in Fig. 4b. Because the energy difference between the photons’ combined energy and the first electronic excited state is ~25,000 cm^−1^, a two-photon absorption mechanism, like that seen in ZnTPP, is impossible. Figure 4b shows two other possible ways that SEVO and ISO may interact with the entangled photons. The first photon must create a coupling with a virtual state. The second photon can then induce a stimulated one-photon scattering back to the ground state, or it can create a coupling with a second virtual state and induce a two-photon scattering. As explained above, these virtual state couplings are very weak because the detuning energy with real eigenstates is very small, particularly in SEVO and ISO which will have detuning energies twice as large as that for ZnTPP. These large detuning energies make either of the mechanisms in Fig. 4b have a very low probability, so any optical signal seen through these mechanisms will be extremely small. As expected, the entangled two-photon interaction signal for SEVO and ISO are 5 and 6 orders of magnitude smaller than the signal from ZnTPP, respectively. In order for SEVO and ISO to have a nonlinear interaction with the entangled photons as described in Fig. 4b, the ethers must have vibrational modes that are active to these nonlinear interactions. To confirm this, we analyzed the neat liquids with Raman spectroscopy, particularly in the low frequency shift “fingerprint” region, with spectra shown in Fig. 5. Both SEVO and ISO have many Raman-active modes in the low frequency shift region, unlike diethyl ether shown for comparison. Another experimental study^15^ has previously shown SEVO and ISO to have active modes in coherent anti-Stokes Raman scattering (CARS), another nonlinear interaction mechanism. While our Raman study and the CARS study^15^ used classical photons, it has already been shown theoretically that entangled photons can be used to induce Raman and other nonlinear interactions^51,52^. Additionally, it has already been shown experimentally by Upton *et al*.^46^ that the use of entangled photons for nonlinear interactions can offer an enhancement in the signal compared to using classical photons. Our experimental results show a similar result: since the entangled photon experiment shows an interaction signal while the classical photon experiment does not show a signal, SEVO and ISO gain an enhancement in their interaction with the use of entangled photons, suggesting that the two anesthetics have a preference for interacting with the quantum system of photons as opposed to the classical system of photons.

Because the experiment is extremely sensitive and can measure very small signals, it is important to ensure that the signal we measure is from SEVO and ISO, not any impurity that may be in the sample. Both neat SEVO and neat ISO were analyzed using mass spectrometry. The mass spectra, shown in Supplementary Figs S6–S7, were compared with literature spectra^53,54^ and show no signs of impurities. (Note, an additional peak at 39.9 m/z in Fig. S6 for ISO is attributed to Ar gas leaking into the instrument during analysis.) We can rule out any possibility of an impurity interacting with the entangled photons.

Given how sensitive the entangled two-photon experiment is to the small signals from SEVO and ISO, it must also be emphasized that this interaction signal seen in SEVO and ISO is not common and cannot be found in just any organic compound. Other organic solvents, such as methanol and toluene, show no signal in this experimental set-up. We also compared the halogenated ethers with another ether that is nonhalogenated, diethyl ether, which showed no signal. The ability to interact with entangled photons through a far-off-resonance transition is a property that is unique to SEVO and ISO and not found in any other organic compounds studied to date.

## Discussion

Although previous studies on the mechanism of action of anesthetics have focused on anesthetics’ ability to interact with macromolecules such as lipid bilayers and proteins, our results show that individual anesthetic molecules can interact with photons. Even more significant is that the halogenated ethers studied here selectively interacted with a quantum system, entangled photons, as opposed to the classical system of photons. This distinction is important because the interaction mechanisms with classical vs entangled photons are different. In both two-photon interactions, the molecule interacting with the photons can take different pathways to go from its initial state to its final state. In classical two-photon interactions, the pathways cannot be controlled, due to the Fourier uncertainty relationship between frequency and time^55^. However, in entangled two-photon interactions, specific pathways can be selected because the entangled photons are not limited by the Fourier uncertainty principle^55^. In other words, during the interaction event with two entangled photons, the molecule and the photons become correlated such that the specific interaction pathway in the molecule is strongly correlated with the quantum state of the photons^55^. In order for a molecule to interact with entangled photons, it must be able to interact with the strongly correlated photons such that it enters into a correlation with them and thus affects the state of the photons; in this case, the molecule affects the photons by interacting with them instead of allowing them to transmit through the medium. Fig. 3 clearly shows that the anesthetics have the ability to interact with the strongly correlated 800 nm entangled photons in our experiment. This is the first empirical evidence that certain anesthetic molecules can directly interact with particles through a quantum mechanism. In addition, diethyl ether, a structurally similar ether, does not interact with the 800 nm entangled photons (transmission plots provided in Supplementary Figs S8–11). Thus, SEVO and ISO have a unique property that diethyl ether does not have. Although not explored in this work, this property may be related to the specific actions of SEVO and ISO vs diethyl ether. We have only investigated the interaction the ethers have with one wavelength of entangled photons, 800 nm. It is not known whether the ethers studied here can or cannot interact with entangled photons of other wavelengths, and this would be an interesting direction for further study.

This result does not prove any mechanism of anesthetic-induced unconsciousness, but it serves as a first proof-of-principle demonstration that anesthetic molecules meet the initial conditions we have outlined to be necessary for a quantum interaction mechanism that may induce unconsciousness. This result motivates further studies on the possible quantum interactions that anesthetic molecules may have in the brain and the atomic or subatomic particles they may be targeting, such as their interaction with the dynamic electronic states of proteins known to be targeted by general anesthetics^56^.

Methodologic strengths of this study include a well-defined experimental model that can differentiate quantum vs. non-quantum interactions as well as the use of both halogenated and non-halogenated ethers. Methodologic limitations include the purely *in vitro* experiments, the high concentration of the anesthetics used, the fact that the anesthetic was in liquid rather than gaseous phase, and the lack of spectral resolution. These limitations make it impossible to determine if there is any neurobiological or clinical relevance associated with the resultant data. Furthermore, the data do not suggest that general anesthesia has an exclusively quantum basis because diethyl ether is an effective anesthetic but demonstrated no quantum interactions. However, we have not conducted a systematic study of all possible quantum interactions with photons, including interaction with other entangled photon wavelengths, or other physical substrates such as microtubules, which have been posited to mediate quantum effects of general anesthetics^2,5^. Further studies should explore other quantum systems as well as more neurobiologically relevant models.

In conclusion, this is (to our knowledge) the first experimental study to rigorously assess the ability of a general anesthetic to interact with subatomic particles with quantum vs. classical features. The finding that halogenation confers the ability of an ether to engage in quantum mechanical processes advances our understanding of these critically important drugs and motivates further investigation regarding non-classical mechanisms of action.

## Methods

### Computational methods

Ground state and first excited state electronic structure calculations were completed using GAMESS^57^. Optimization of ground state geometries and calculation of molecular orbitals were completed with DFT using the B3LYP functional and 6–311 + G(2d,p) basis set. A previous study confirmed the accuracy of this functional and basis set for SEVO and another inhaled anesthetic, halothane^27^. We compared our optimized geometries, ground state dipole moments, and molecular orbitals with previous studies to confirm the accuracy of our calculations^18,20–22,27,45^. Images of molecular orbitals in Supplementary Figs S1–S5 were created using wxMacMolPlt^58^. Electronic properties of the first excited state, including the excitation energy, transition dipole moment, oscillator strength, and TPA cross-section, were completed with TD-DFT using the same functional and basis set.

### Materials

Sevoflurane (C_4_H_3_F_7_O) and isoflurane (C_3_H_2_ClF_5_O) were obtained commercially and used without further purification methods. No impurities were found in their respective mass spectra (See Supplementary Figs S6–S7). HPLC grade methanol (≥99.9%) and diethyl ether (≥99.9%) were obtained from Sigma-Aldrich and used as received.

### Steady-state spectroscopy

UV-Vis absorption spectra (190–900 nm) were measured on an Agilent 8432 UV−visible spectrometer. Fluorescence spectra were collected on a Varian Cary Eclipse fluorimeter. 1.0 cm pathlength quartz cuvettes were used for all measurements.

### Classical two-photon excited fluorescence (TPEF)

The two-photon excited fluorescence technique^59,60^ was used for the classical two-photon experiments. Our experimental setup has been described elsewhere^49,61^. In brief, the pure liquid (or a liquid solution of the compound of interest) contained in a 1 cm path length quartz cuvette is excited with the output beam of a Ti:Sapphire femtosecond pulsed laser (KMLabs, 800 nm, Δt ~30 fs) with a 80 MHz repetition rate. The fluorescence emission was collected at a 90° angle to the excitation beam. Fluorescence emission at a selected wavelength was detected using a monochromator (ORIEL, Cornerstone 130) and a photomultiplier tube (Hamamatsu). The power of the 800 nm excitation beam was changed by using a variable neutral density filter.

### Entangled two-photon spectroscopy

**Figure 6:**
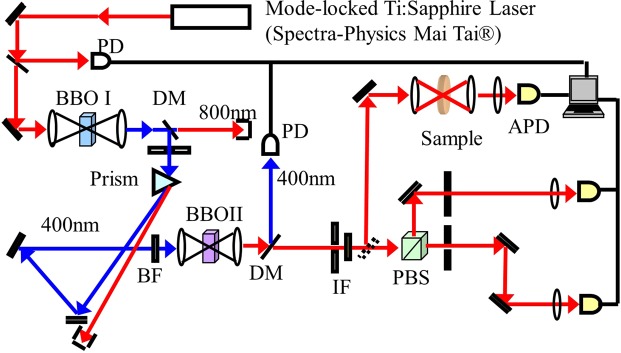
Experimental setup used for entangled two-photon spectroscopy.

The entangled two-photon spectroscopy technique has been previously described^39,46,48,49^. A sketch of the experimental setup used in this work is presented in Fig. 6. Orthogonally polarized entangled photon pairs were generated by the spontaneous parametric down-conversion (SPDC) process. A 0.5 mm BBO (*β*-Barium Borate) crystal (type II) is pumped with the second harmonic generation (SHG) beam, 400 nm, of a Ti:Sapphire pulsed laser emitting ~70 fs pulses (MaiTai, Spectra Physics). Entangled photon intensity is varied by changing the pump power on the SPDC crystal with a variable neutral density filter. Transmitted entangled photons are focused onto an avalanche photodiode (SPCM-AQR13, PerkinElmer). Fig. 6 shows the complete set-up, previously shown by Harpham *et al*.^48^.

## Supplementary information

Supplementary Information

## Data Availability

The data that support the findings of this study are available from the corresponding author upon reasonable request.
