# Author Correction: Modern Anesthetic Ethers Demonstrate Quantum Interactions with Entangled Photons

**DOI:** 10.1038/s41598-021-88469-0

**Published:** 2021-04-20

**Authors:** Ryan K. Burdick, Juan P. Villabona-Monsalve, George A. Mashour, Theodore Goodson

**Affiliations:** 1 Department of Chemistry, University of Michigan, Ann Arbor, MI 48109 USA; 2 Center for Consciousness Science, Department of Anesthesiology, University of Michigan Medical School, Ann Arbor, MI 48109-5048 USA

Correction to: *Scientific Reports* 10.1038/s41598-019-47651-1, published online 05 August 2019

The Acknowledgements section in the original version of this Article was incomplete.

“This work was based on work supported by the National Science Foundation through Grant CHE 1607949 (TGIII).”

now reads:

“This work was based on work supported by the National Science Foundation through Grant CHE 1607949 (TGIII). This material is based upon work supported by the Air Force Office of Scientific Research under award number FA9550-17-1-0457."

The original Article has been corrected.

