# Supplementary material for: Modern Anesthetic Ethers Demonstrate Quantum Interactions with Entangled Photons
Source: Sci Rep. 2019 Aug 5;9:11351. doi: 10.1038/s41598-019-47651-1 (PMC6683176; doi:10.1038/s41598-019-47651-1)
Supplement: Supplementary file 1 — Supplementary Information [file 41598_2019_47651_MOESM1_ESM.pdf]

Modern Anesthetic Ethers Demonstrate Quantum Interactions with Entangled Photons

Ryan K. Burdick, [†] Juan P. Villabona-Monsalve,[†] George A. Mashour[‡,**] and Theodore Goodson III. [†,*]

[†]Department of Chemistry, University of Michigan, Ann Arbor, MI 48109

[‡] Center for Consciousness Science, Department of Anesthesiology, University of Michigan Medical School, Ann Arbor, MI 48109-5048

[*]tgoodson@umich.edu, [**]gmashour@med.umich.edu

Supplementary Information

Supplementary Fig. S1-S5 show the HOMO and LUMO of methyl isopropyl ether, SEVO, ethyl methyl ether, ISO, and diethyl ether, respectively. Isosurface values were chosen such that only the most predominant location of each orbital is shown in order to identify the character of each orbital. The orbitals for SEVO in Supplementary Fig. S2 show a $n_O \rightarrow \sigma_{C\text{-}H}^*$ HOMO-LUMO transition, and the orbitals for ISO in Supplementary Fig. S4 show a $n_{Cl} \rightarrow \sigma_{C\text{-}Cl}^*$ HOMO-LUMO transition, both of which match previously reported orbital characteristics for SEVO[3,4] and ISO[5], respectively. While the orbitals for SEVO and ISO have been reported before, we show our results here as further confirmation of the accuracy of our electronic structure calculations.

Supplementary Fig. S6-S7 show the mass spectra of the SEVO and ISO, respectively, used in our experiments. Mass spectra were obtained through electron ionization using a Micromass AutoSpec Ultima magnetic sector mass spectrometer.

Because the laser power in the entangled two-photon spectroscopy set-up can fluctuate in a short period of time, each time a new solution is to be tested, a fresh solvent transmission scan must be completed before the transmission scan of the new solution. This method ensures the highest accuracy when comparing a solution's transmission line to a solvent's transmission line to determine whether or not the solution is interacting with entangled photons. When completing

these two scan back-to-back, the random fluctuations of the laser's power should be minimized, as opposed to comparing a solution's transmission lines to a solvent transmission line taken hours earlier. Each of the transmission lines for the solutions tested in Supplementary Fig. S8-S11 are compared with the solvent transmission line acquired immediately before the solution was tested. The pure liquids SEVO and ISO were tested, with each solution's transmission lines (and solvent transmission lines of pure methanol) shown in Supplementary Fig. S8 for SEVO and Supplementary Fig. S9 for ISO. The same experiment and analysis were completed for diethyl ether and methanol, shown in Supplementary Fig. S10. Zinc tetraphenylporphyrin (ZnTPP) in toluene was used as a standard to validate our experiment and analysis, shown in Supplementary Fig. S11.

Supplementary Fig. S11 shows a difference between the transmission lines for ZnTPP and toluene, which is expected since this compound has previously been reported to interact with entangled photons[6,7], thus validating our experiment and analysis. Supplementary Fig. S10 shows that the transmission lines for diethyl ether and methanol lie within each other's error bars. Therefore, interaction is not occurring in diethyl ether, and the difference in the transmission lines is due to random fluctuations of the laser. However, SEVO in Supplementary Fig. S7 and ISO in Supplementary Fig. S8 have transmission lines outside of the error bars of their respective solvent transmission lines. Therefore, these two samples are interacting with entangled photons.

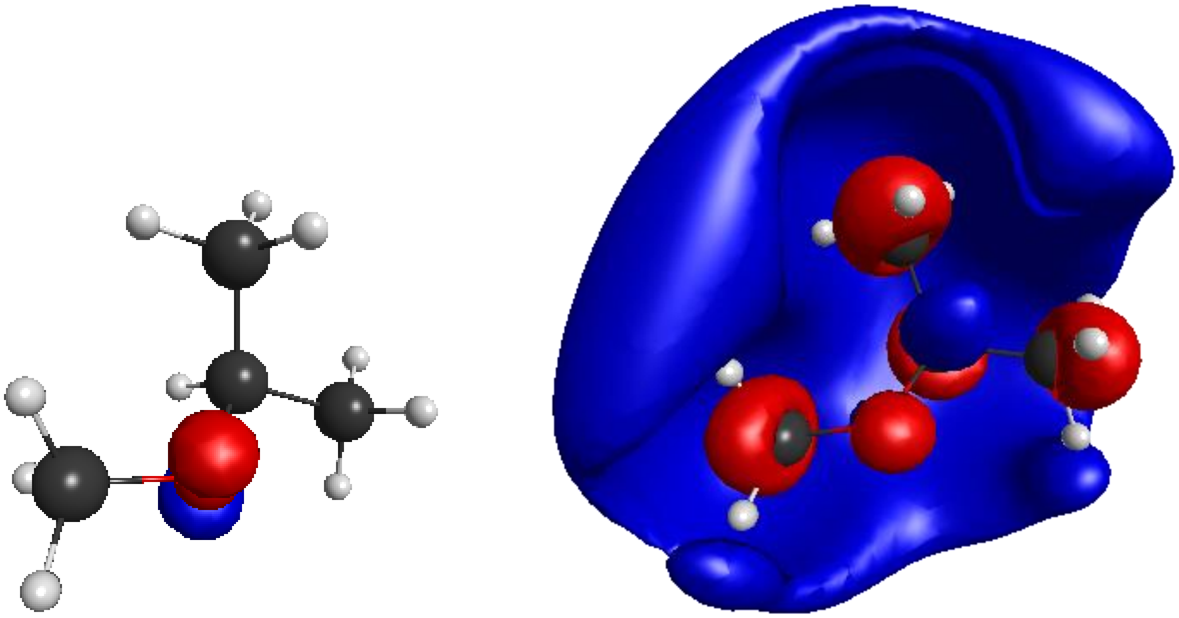

Supplementary Figure S1. HOMO (left) and LUMO (right) of the ground state (S_0) of methyl isopropyl ether.

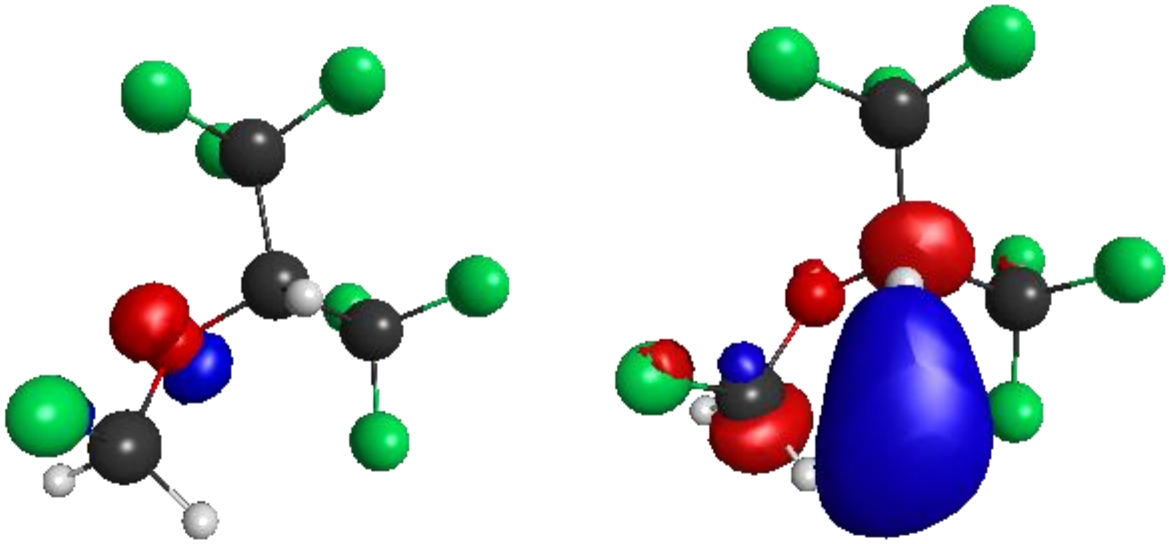

Supplementary Figure S2. HOMO (left) and LUMO (right) of the ground state (S_0) of SEVO.

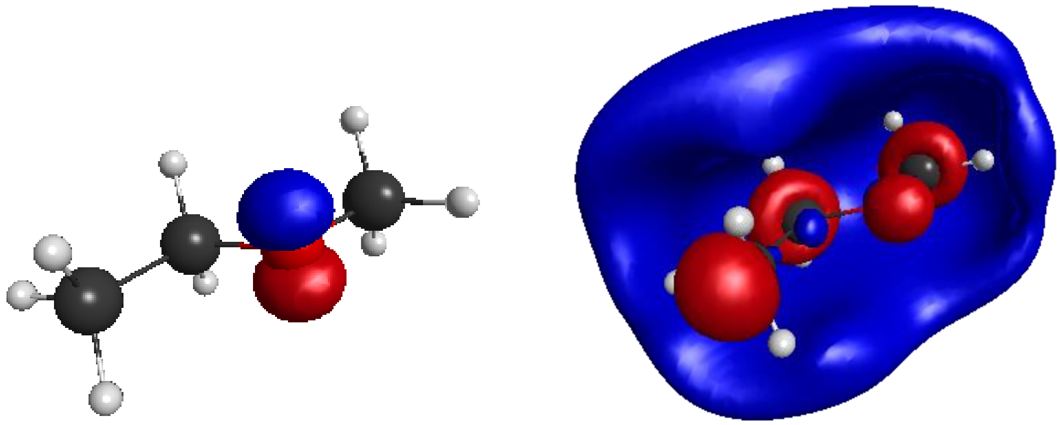

Supplementary Figure S3. HOMO (left) and LUMO (right) of the ground state (S_0) of ethyl methyl ether.

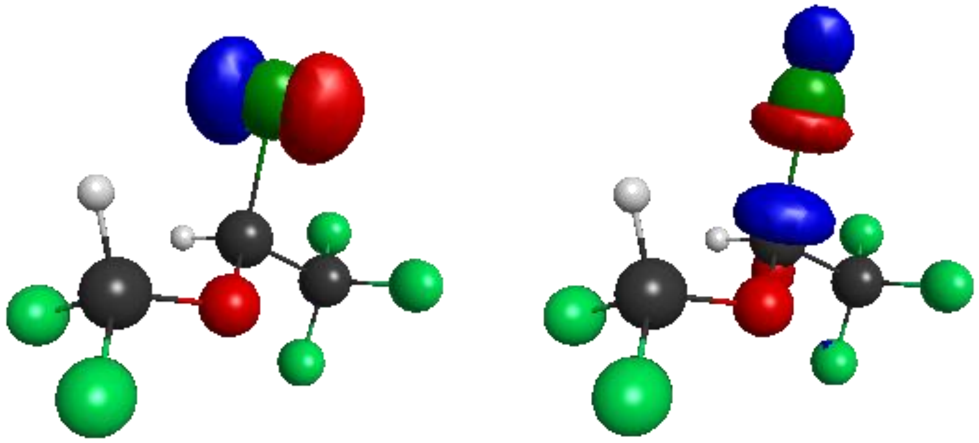

Supplementary Figure S4. HOMO (left) and LUMO (right) of the ground state (S_0) of ISO.

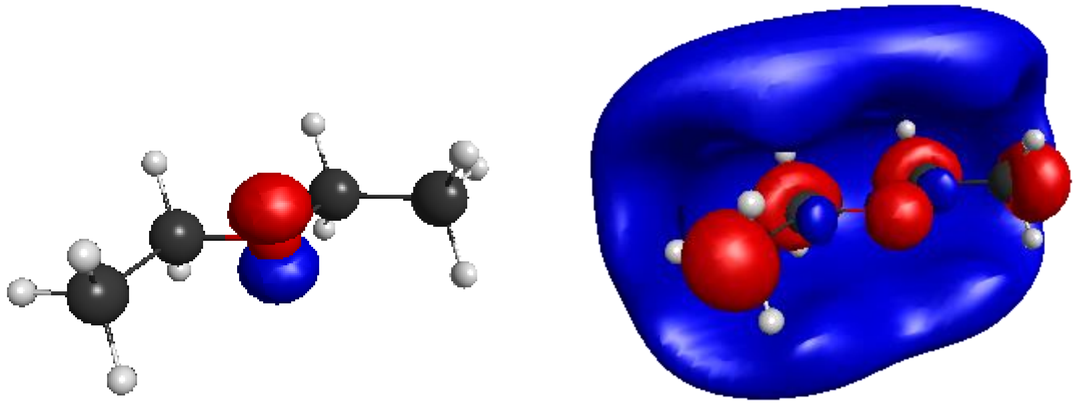

Supplementary Figure S5. HOMO (left) and LUMO (right) of the ground state (S_0) of diethyl ether.

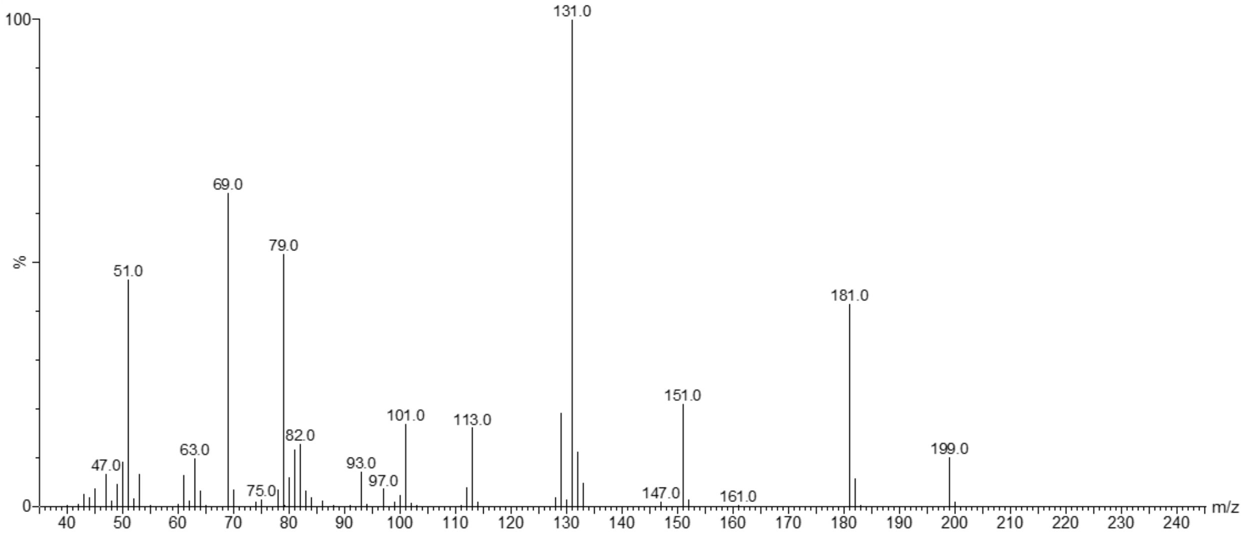

Supplementary Figure S6. Mass spectrum of SEVO.

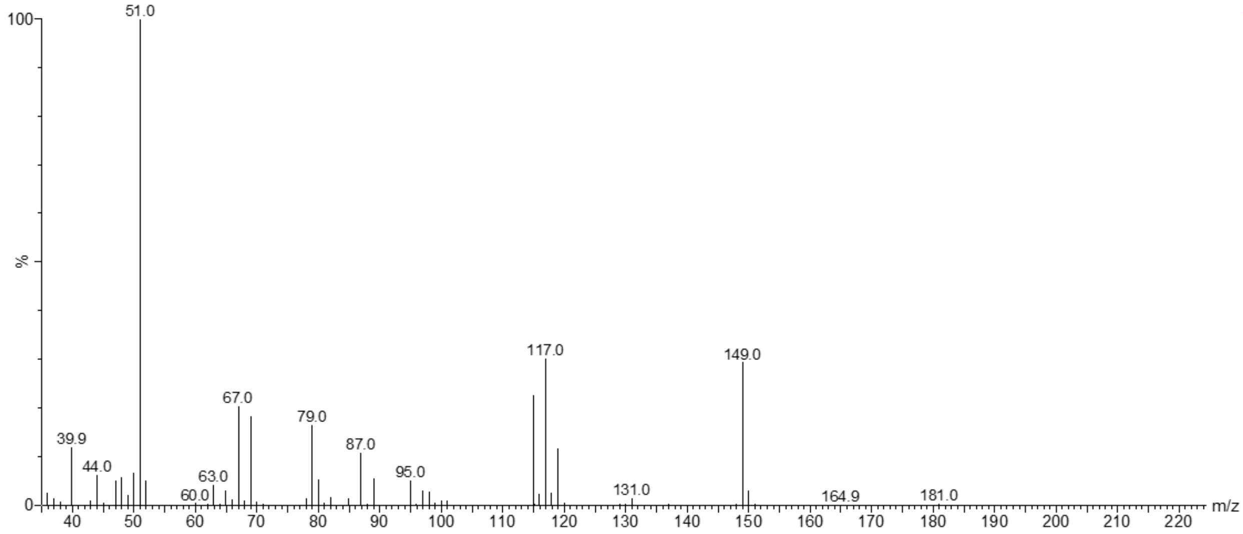

Supplementary Figure S7. Mass spectrum of ISO.

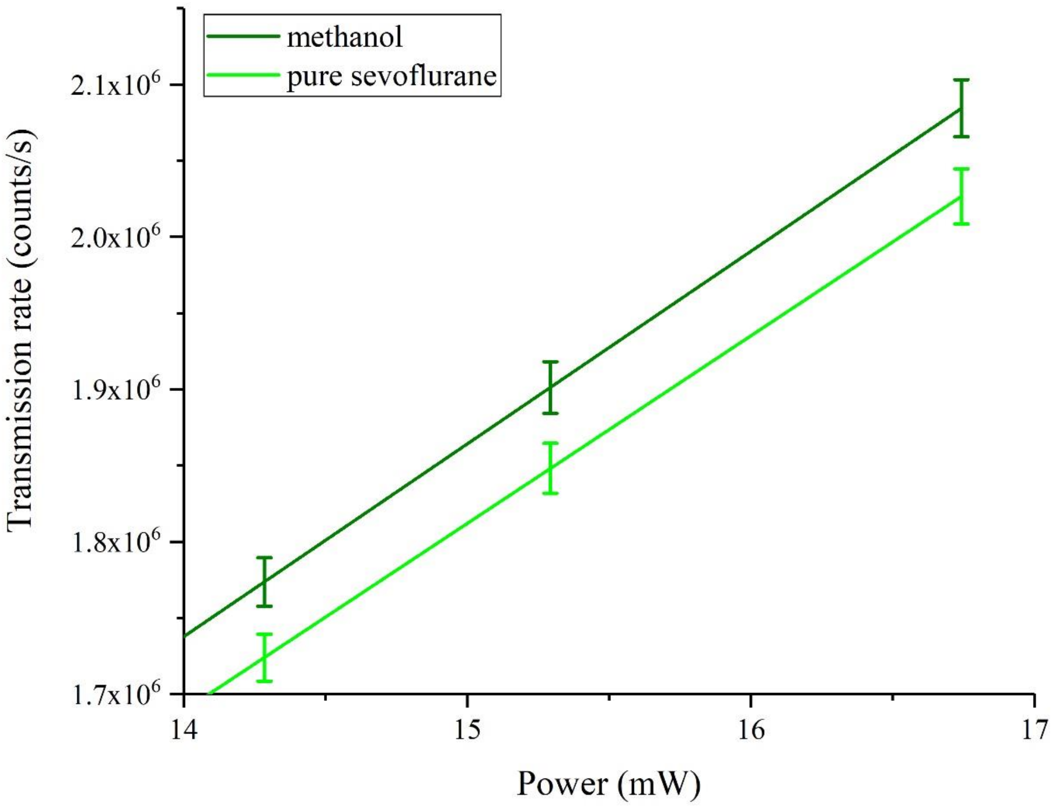

Supplementary Figure S8. Transmission rate as a function of incident laser power for pure SEVO, compared to methanol.

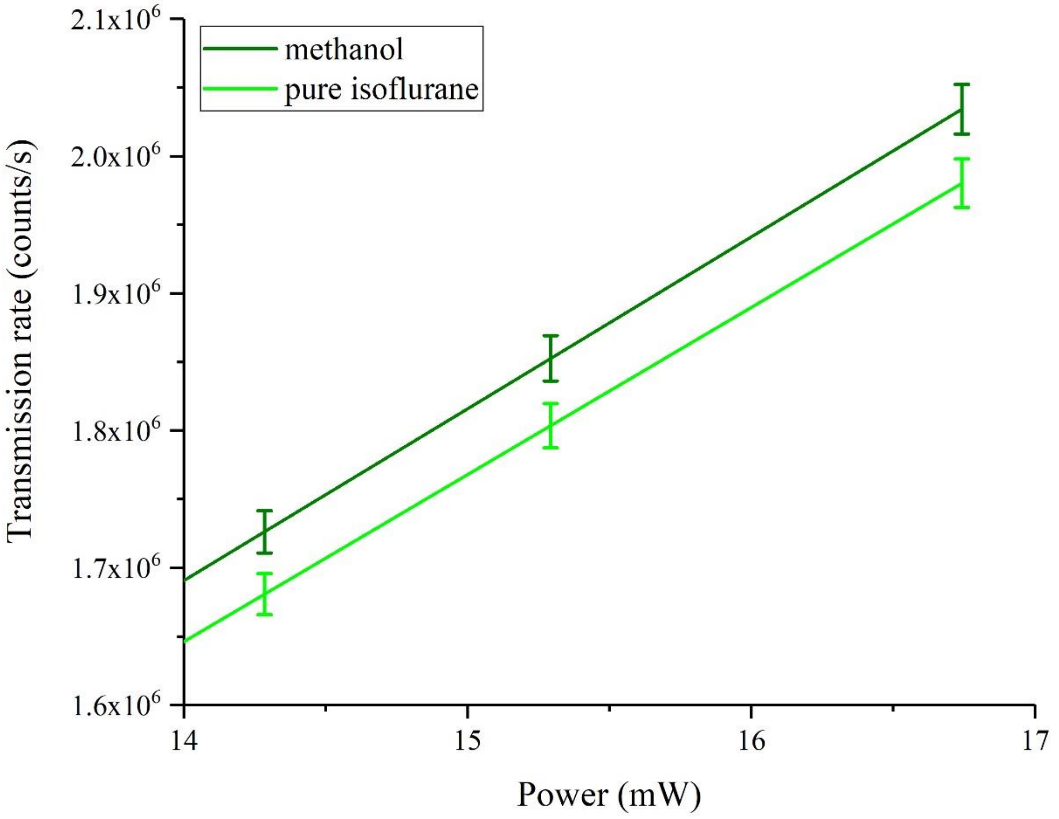

Supplementary Figure S9. Transmission rate as a function of incident laser power for solutions of ISO, compared to methanol.

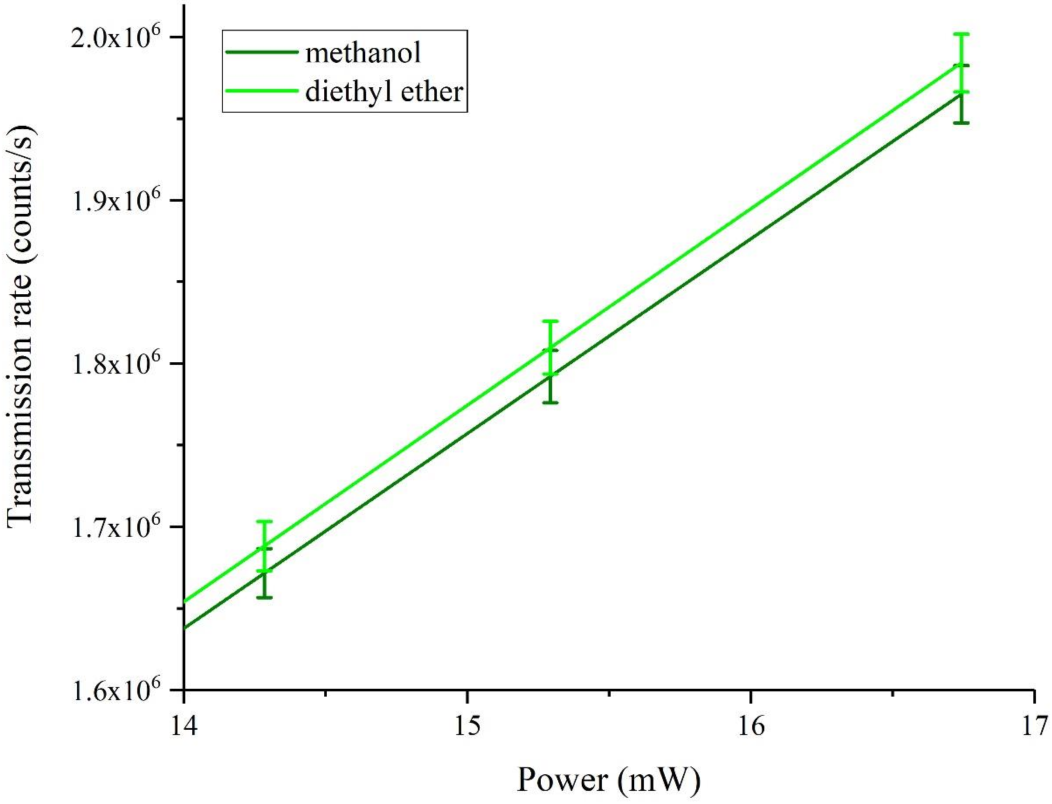

Supplementary Figure S10. Transmission rate as a function of incident laser power for diethyl ether, compared to methanol.

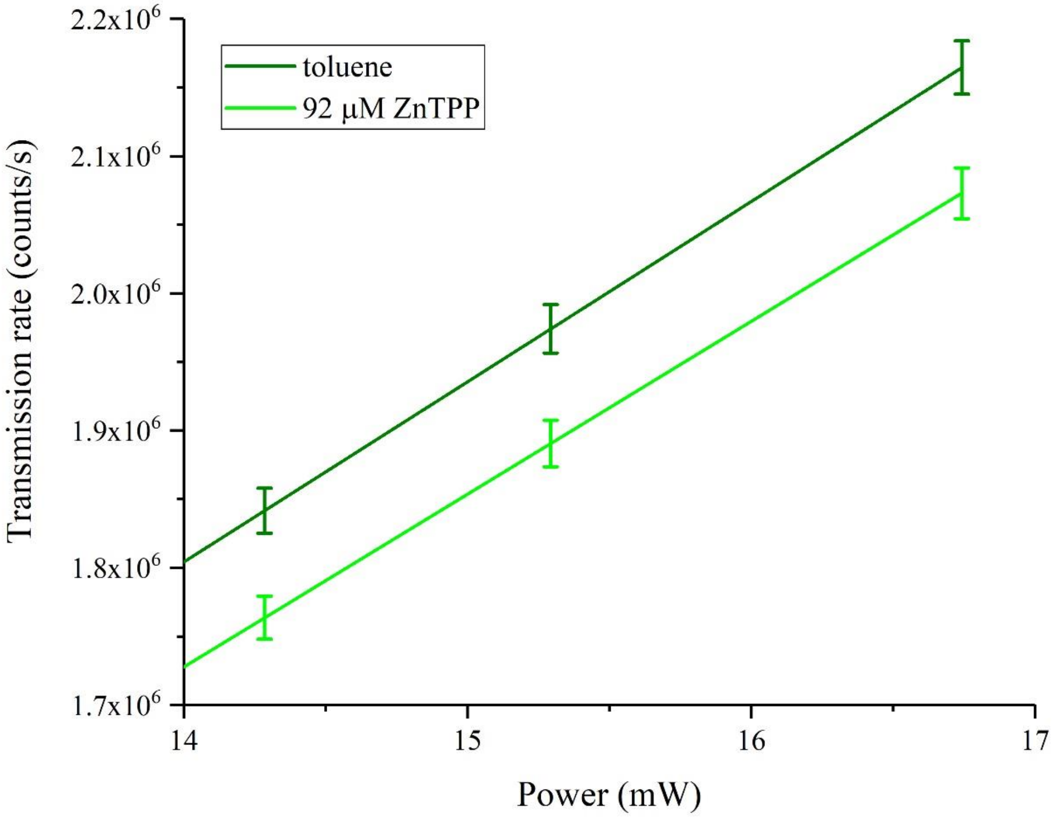

Supplementary Figure S11. Transmission rate as a function of incident laser power for ZnTPP in toluene, compared to toluene.

References

[1] Bouche, M.-P. L. A. *et al.* Quantitative determination of vapor-phase compound a in sevoflurane anesthesia using gas chromatography–mass spectrometry. *Clin. Chem.* **47**, 281 (2001).

[2] Deng, X.-s. & Simpson, V. J. Determination of volatile anesthetics isoflurane and enflurane in mouse brain tissues using gas chromatography-mass spectrometry. *J. Pharmacol. Toxicol. Methods* **49**, 131-136, https://doi.org/10.1016/j.vascn.2003.11.002 (2004).

[3] Freitas, M. P., Bühl, M., O'Hagan, D., Cormanich, R. A. & Tormena, C. F. Stereoelectronic interactions and the one-bond c–f coupling constant in sevoflurane. *J. Phys. Chem. A* **116**, 1677-1682, 10.1021/jp211949m (2012).

[4] Tang, P., Zubryzcki, I. & Xu, Y. Ab initio calculation of structures and properties of halogenated general anesthetics: Halothane and sevoflurane. *J. Comput. Chem.* **22**, 436-

444, https://doi.org/10.1002/1096-987X(200103)22:4<436::AID-JCC1014>3.0.CO;2-U (2001).
[5] Lesarri, A. *et al.* Structural evidence of anomeric effects in the anesthetic isoflurane. *PCCP* **13**, 6610-6618, 10.1039/C0CP02465A (2011).
[6] Villabona-Monsalve, J. P., Calderón-Losada, O., Nuñez Portela, M. & Valencia, A. Entangled two photon absorption cross section on the 808 nm region for the common dyes zinc tetraphenylporphyrin and rhodamine b. *J. Phys. Chem. A* **121**, 7869-7875, 10.1021/acs.jpca.7b06450 (2017).
[7] Upton, L. *et al.* Optically excited entangled states in organic molecules illuminate the dark. *J. Phys. Chem. Lett.* **4**, 2046-2052, 10.1021/jz400851d (2013).
